# On the Cause and Treatment of Abortion and Sterility, &c.

**Published:** 1849-01

**Authors:** 


### ARTICLE V.

*On the Cause and Treatment of Abortion and Sterility; being the result of an extended Practical Inquiry into the Physiological and Morbid Conditions of the Uterus, with reference especially to Leucorrhœal Affections, and the diseases of Menstruation.* By JAMES WHITEHEAD, F. R. C. S., Surgeon to the Manchester and Salford Lying-in Hospital. pp. 368. Philadelphia, Lea & Blanchard, 1848. (For sale by J. Keen & Bro., Chicago.)

This is the fullest and clearest account of these affections that has come to our notice. The work is full of interest to the practitioner, as embodying a large mass of physiological and pathological facts, and the report of many cases of disease, showing the results of treatment.

We shall endeavor to give a more extended notice of it in our next. In the mean time, we have no hesitation in advising our professional brethren to buy and attentively read the work.

